# Supplementary material for: The interactome of intact mitochondria by cross-linking mass spectrometry provides evidence for coexisting respiratory supercomplexes
Source: Mol Cell Proteomics. 2017 Dec 8;17(2):216–32. doi: 10.1074/mcp.RA117.000470 (PMC5795388; doi:10.1074/mcp.RA117.000470)
Supplement: Supplemental Data [file supp_RA117.000470_133922_0_supp_23978_fzffwf.zip › spectra_annotation/mito_spectra_annotation/483-1-4-1-2-1.pdf]

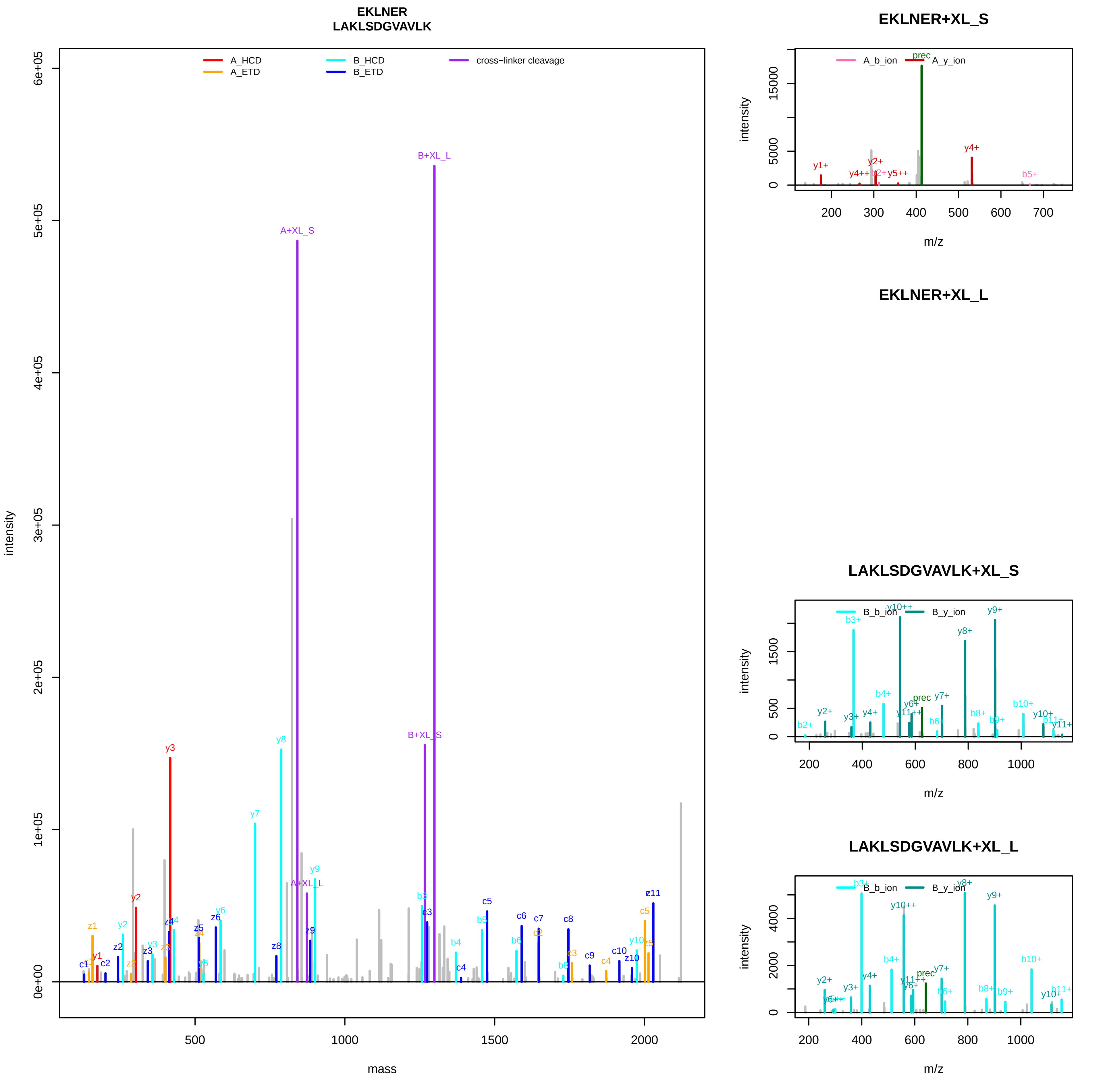

EKLNER
LAKLSDGVAVLK
A_HCD
A_ETD
B_HCD
B_ETD
cross−linker cleavage
intensity
mass
0e+00
1e+05
2e+05
3e+05
4e+05
5e+05
6e+05
500
1000
1500
2000
B+XL_L
A+XL_S
B+XL_S
A+XL_L
y3
y2
y1
z1
y2
z2
c2
c1
z3
y3
z4
y4
z5
z6
y6
y7
y8
y9
z8
z9
b3
c3
b4
c4
c5
b5
b6
c6
c7
c8
b8
c3
c9
c4
c10
z10
y10
c5
z11
EKLNER+XL_S
A_b_ion
A_y_ion
prec
y1+
y2+
y4++
y5++
y4+
b5+
m/z
EKLNER+XL_L
LAKLSDGVAVLK+XL_S
B_b_ion
B_y_ion
y10++
y9+
b3+
y8+
b4+
y2+
y3+
y4+
y6+
y11++
prec
y7+
b6+
b8+
b9+
b10+
y10+
b11+
y11+
b2+
LAKLSDGVAVLK+XL_L
b3+
y8+
y9+
y10++
b4+
y4+
y2+
y3+
y11++
y7+
y6+
b6+
b8+
b9+
b10+
y10+
b11+
